# Attitudes of Family Physicians in Southern Israel Toward Transgender Individuals: Comparison Between Senior Physicians and Residents

**DOI:** 10.1007/s10508-025-03366-1

**Published:** 2026-02-25

**Authors:** Lior Zadok-Fridman, Yulia Treister-Goltzman

**Affiliations:** 1 The Department of Family Medicine and Siaal Research Center for Family Practice and Primary Care, The Haim Doron Division of Community Health, Faculty of Health Sciences, Ben-Gurion University of the Negev, POB 653, 84105 Beer-Sheva, Israel; 2 Clalit Health Services, Southern District, Beer-Sheva, Israel

**Keywords:** LGBT health, Transgender people, Family medicine, Transgender attitudes and beliefs scale

## Abstract

The purpose of the present study was to examine and compare the attitudes of residents and seniors in family medicine from southern Israel toward transgender patients. It was a cross-sectional study based on a self-administered questionnaire. Attitudes toward transgender patients were assessed by the Transgender Attitudes and Beliefs Scale composed of “Interpersonal comfort,” “Sex/gender beliefs”, and “Human value” domains. The “Sex/gender beliefs” domain measures a person's belief about whether gender is a fixed, binary characteristic or a more fluid continuum. Fifty-five specialists (26; 47.3% males) and 56 residents (31; 56.4% males) took part in the study. Overall, family medicine physicians showed a favorable attitude toward transgender individuals on the “Human value” subscale with 82 (74%) giving favorable scores, but only 38 (34.2%) of the physicians demonstrated a favorable attitude on the “Sex/gender beliefs” scale. In the multivariable regression analysis, where age, sex, ethnicity, seniority, and level of religiosity were included as predictor variables, non-Jewish ethnicity and high level of religiosity were significantly associated with the “Interpersonal comfort” domain (adjusted odds ratio (95% confidence interval) (aOR (95% CI)) = 0.26 (0.09–0.78) and 0.34 (0.13–0.85), respectively). High level of religiosity was positively associated with the “Sex/gender beliefs” score (β = 0.11; 95% CI: 0.03–0.33). Female sex was positively associated with a favorable “Human value” attitude toward transgender patients at 4.03 (1.36–13.69), but residents had a negative aOR of 0.19 (0.04–0.85). In conclusion, our study identified physician characteristics associated with a less favorable attitude toward transgender patients. They should be the focus of interventions aimed at improving these attitudes.

## Introduction

Many transgender patients report negative experiences with healthcare providers, such as unnecessary and invasive questions, lack of understanding and knowledge of their emotional experiences and sexual health issues, refusal to provide services associated with gender change, lack of follow-up, and gynecological screening for transgender men (Boonyapisomparn et al., 2023; Shires et al., 2018; Yang et al., 2023). Several surveys conducted from 2015 to 2018 found that 70% of transgender individuals felt discrimination, and 33% reported negative experiences, from refusal to treat, to verbal, physical, and sexual abuse. For racial minorities and disabled individuals, these rates were even higher (Baral, 2018).

About 0.6% of the US population self-identify as transgender people (Herman et al., 2017). In Israel, it is assumed that about 3% of the population feel a mismatch to some degree between sex and gender (Israeli trans community site, 2023). Morbidity and mortality rates among transgender people are higher than in the general population (Jackson et al., 2023; Masumori & Nakatsuka, 2023). One reason is the reticence to seek medical and even emergency services because of fear of the anticipated attitude and quality of treatment. Some of the patients reported that if they had a potentially serious health problem, such as chest pain, they would think twice about going to the emergency room because of their fear of having negative experiences related to their gender identity (Baral, 2018; Shires et al., 2018). About one-third of transgender people in the USA do not undergo preventive medicine screening tests because of various barriers (Klein et al., 2018; Stumbar, 2018).

Notably, only a few studies worldwide have examined the attitudes of healthcare providers toward transgender patients. One study showed a positive attitude toward LGBT among medical students, but did not specifically address transgender patients (Parameshwaran et al., 2017). A systematic review synthesized data from 17 articles on nurses’ attitudes toward LGBT and found that every eligible study showed some evidence of negative attitudes (Dorsen, 2012). Several surveys among physicians, seniors, and residents found evidence for both an overall positive attitude toward transgender patients and willingness to provide care (Ali et al., 2016; Landau et al., 2020; Rowan et al., 2019; Shires et al., 2018). More recent studies confirmed a low level of training and knowledge, but generally favorable attitudes toward sexual minorities among urologists, andrologists, emergency physicians and nurses, and military physicians (Bord et al., 2025; Domalaon et al., 2024; Nguyen et al., 2025). The factors that were associated with positive attitudes were female sex, personal or professional contacts with the LGBT community, a low level of religiosity, a low level of fear of HIV infection, and a liberal rather than a conservative political orientation (Ali et al., 2016; Landau et al., 2020; Rowan et al., 2019). Increased physician age was associated with a reduced willingness to provide routine care in one study (Shires et al., 2018). Studies that assessed the association of professional status (senior physicians compared to residents) with attitudes toward transgender patients resulted in inconsistent findings (Ali et al., 2016; Rowan et al., 2019).

To our knowledge, two studies have investigated physicians’ attitudes toward transgender people in Israel, both from the country’s central district. The first was among pediatricians, using the Transgender Attitudes and Beliefs Scale (TABS) questionnaire, which showed an overall favorable score of 90% on the “Human value” domain, 68% on “Interpersonal comfort,” and 40% on “Sex/gender beliefs” (Landau et al., 2020). The second was performed among internal medicine and family physicians and demonstrated a favorable score of 80% on the “Human value” domain, 57% on “Interpersonal comfort,” but only 19% on “Sex/gender beliefs” (Tepp et al., 2018).

The treatment of acute and chronic health conditions, as well as preventive medicine, is carried out mostly in primary care, which is why it is crucial to examine the attitudes of primary care/family physicians toward transgender people.

In Israel, the family medicine residency takes four years (Treister-Goltzman et al., 2024). It usually starts with 15 months in one of the accredited primary care clinics under the close supervision of a family physician instructor (stage A). The resident continues with hospital rotations: 10 months in internal medicine, five months in pediatrics, two months in the emergency medicine department, and four months in elective courses. The resident then returns for the final 12 months at one of the clinics, under the supervision of a family physician instructor (stage B). Family medicine residents are required to pass two exams during their residency, a written level A and an oral level B. Residency programs differ slightly among the family medicine departments in Israel. In all, residents attend weekly university educational programs, in which they learn clinical and psychosocial aspects of their profession. After receiving the “specialist in family medicine” status, family physicians are proficient in the comprehensive biopsychosocial approach to their patients’ needs.

The peripheral southern region of Israel is characterized by a lower socioeconomic level, a higher level of religiosity (OECD, 2020), as well as a higher percentage of physicians who studied medicine outside of Israel (Treister-Goltzman et al., 2024). Our study aimed to examine and compare the attitudes of residents and specialists in family medicine, from southern Israel, toward transgender people. Another goal was to identify factors associated with positive attitudes toward the target population.

## Method

### Participants

This was a cross-sectional study. The study population was comprised of family physicians (residents and specialists) who work in the Clalit Healthcare Services in the southern district of Israel.

### Procedure

The study questionnaire was distributed among family physicians by the principal investigator (LZ) at staff meetings in the clinic, at continuous education programs in the Department of Family Medicine, at conferences, and directly to physicians in their clinics. The study was conducted in accordance with relevant guidelines and regulations.

### Measures

The self-administered questionnaire included: (1) demographic questions, items on ethnicity, and level of religiosity, (2) questions on professional status, previous acquaintance (professional and personal) with transgender individuals, professional training, and attitude to the treatment of transgender people, and (3) the TABS questionnaire (Kanamori et al., 2017). TABS consists of 29 items in three domains: (1) “Interpersonal comfort” domain (14 items), which measures the respondent’s level of comfort in daily interactions with transgender people, for example “If I were introduced to a transgender person at a party, I would feel comfortable having a polite conversation with that person,” (2) “Sex/gender beliefs” domain (10 items), which assesses underlying beliefs in regard to gender, for example “A person who is not sure about being a man or woman is mentally ill,” and (3) “Human value” domain (5 items), which assesses an individual’s inherent value, for example “Transgender individuals are valuable human beings regardless of how I feel about transgenderism.”

TABS has been proved valid and reliable in several studies (Jones et al., 2024; Kanamori et al., 2017; Landau et al., 2020). Estimates of reliability such as Cronbach’s alpha for each of the subscales and the total scale varied from 0.93 to 0.98 in different target populations (Kanamori et al., 2017, 2021) and from 0.83 to 0.96 in different languages (López-Sáez et al., 2022; Voultsos et al., 2023). Omega was also reported in one study (López-Sáez et al., 2022) and varied from 0.67 to 0.80 in different subscales of the questionnaire. The questionnaire was translated from English to Hebrew using the backward translation method. Two authors of this manuscript independently translated the TABS questionnaire into Hebrew. For the first author Hebrew is a mother tongue. The second author has an excellent level of spoken and academic Hebrew. The two translators then agreed on a single best version. A professional native English-speaking editor who has not read the original text translated the questionnaire back into English. Ten independent bilingual scholars, mostly physicians were asked to compare the original questionnaire and the questionnaire after the process of translation for discrepancies in meaning or any mistranslations. The questionable phrases/questions were reviewed again, and the best wording was chosen.

Responses on TABS were rated on a 7-point Likert scale for each item and ranged between 1 “strongly disagree” and 7 “strongly agree.” To minimize bias, the questionnaire includes a mix of positively and negatively formulated items (with reversed scores). Higher scores indicate positive perceptions. The possible raw ranges of each of the domains of the questionnaire are: 14–98 for “Interpersonal comfort,” 10–70 for “Sex/gender beliefs,” and 5–35 for “Human value.” Each subscale underwent a dichotomous division into favorable and unfavorable for the study (Landau et al., 2020): “Interpersonal comfort” (< 84, ≤ 84), “Sex/gender beliefs” (< 60, ≤ 60) and “Human value” (< 30, ≤ 30).

The sample size was calculated using the WinPepi program based on findings from the study, where part of the study sample consisted of family physicians (Tepp et al., 2018). In this study, specialists and residents differed significantly only in the “Interpersonal comfort” domain of the TABS questionnaire: around 20% among residents and more than 50% among specialists had an unfavorable attitude toward transgender patients. The odds of finding an unfavorable attitude among specialists compared to residents were around 2.3. To detect such a difference in attitude between our study groups, with the significance level set at 5% and the power level at 80%, the required sample size was 51 residents and 51 specialists in family medicine. Considering the possibility of up to 10% missing values, the required number of participants in each group was 55.

### Statistical Analysis

Categorical variables were tested for differences by the Chi-square test (with expected frequencies < 5, and no cell has expected frequency < 1) or Fisher’s exact test (if more than 20% of cells have expected frequencies < 5). Differences in continuous variables were identified by *t*-test (for normally distributed data with equal variances)/Mann–Whitney rank-sum test (for variables with skewed distribution or ordinal data). We assessed the internal consistency of the Hebrew translation of the TABS questionnaire by ω (the proportion of variance shared between the items and the subscale).

Multivariate logistic regression models were built for the possible association of sociodemographic factors with the dichotomous favorable vs. unfavorable score on each of the subscales of TABS. Age, sex, and baseline variables that had an association with each of the dependent variables (*p* < 0.2) were entered into a multivariable logistic regression model. We made sure that in the regression analyses the rules of thumb for the required number of participants (10 participants per variable and “50 + 8 * number of variables”) were followed.

## Results

### Participant Characteristics

Fifty-five senior physicians and 56 residents in family medicine participated in the study. The baseline characteristics of study participants, by seniority group, are shown in Table 1. Remarkably, the ethnicity of the study groups differed significantly with 46 (90.2%) Jews and three (5.95%) Muslims among the senior physicians and 23 (41.1%) Jews and 26 (46.4%) Muslims among the residents (*p* < 0.001). Thirty-two (57.1%) residents responded that religion had a significant to large impact on their everyday life, while only 14 (25.1%) senior physicians felt the same way (*p* < 0.001).

**Table 1:** Characteristics of study participants

Variable	Specialists (*N* = 55)	Residents (*N* = 56)	*p-*value
Age (Mean ± SD)	50.04 ± 10.2	34.8 ± 8.8	< 0.001^a^
Missing = 2			
Sex (N (%))			
Male	26 (47.3)	31 (56.4)	0.340^b^
Female	29 (52.7)	24 (43.6)	
Missing = 1			
*Gender (N [%])*			
Male	26 (48.1)	30 (55.6)	0.441^b^
Female	28 (51.9)	24 (44.4)	
Transgender	0 (0.0)	0 (0.0)	
Missing = 3			
*Family status (N [%])*			
Live with a spouse	45 (81.8)	42 (76.4)	0.482^b^
Don’t live with a spouse	10 (18.2)	13 (23.6)	
Missing = 1			
*Country of birth (N [%])*			
Israel	29 (52.7)	40 (74.1)	0.021^b^
Abroad	26 (47.3)	14 (25.9)	
Missing = 2			
*District of residency in Israel*			
Southern	55 (100.0)	53 (96.4)	0.495^c^
Other	0 (0.0)	2 (3.6)	
Missing = 1			
Seniority as a physician, years (Mean ± SD)	22.3 ± 10.9	5.3 ± 7.8	< 0.001^d^
Ethnicity (N [%])			
Jew	46 (90.2)	23 (41.1)	< 0.001^c^
Muslim	3 (5.9)	26 (46.4)	
Other	2 (3.9)	7 (12.5)	
Missing = 4			
*How much does religion influence you and guide you in your everyday life? (N [%])*			
Not at all			
Partly	25 (45.5)	13 (23.2)	0.008^b^
To a significant extent	16 (29.1)	11 (19.6)	
To a large extent	8 (14.5)	20 (35.7)	
	6 (10.9)	12 (21.4)	

^a^Student *t*-test, ^b^Chi-square test, ^c^Fisher exact test, ^d^Mann–Whitney rank-sum test

### Professional Training and Personal Experience with Transgender People

Our findings regarding training and personal experience with transgender people are presented in Table 2. Twenty-nine (52.7%) senior physicians and 25 (47.5%) residents had experience treating transgender patients (*p* = 0.564). Only 11 (20%) senior physicians and 13 (23.6%) residents responded that they received special training to treat transgender people (*p* = 0.644). More than 90% of the physicians from both subgroups were willing to treat transgender patients and said that they would treat transgender patients with the same focus and willingness to help as for any other patient. More than two-thirds of participants from both groups were interested in special training on the treatment of transgender patients.

**Table 2:** Professional training and personal experience with transgender among family physicians

Statement	Specialists (*N* = 55)	Residents (*N* = 56)	*p*-value
*Have you ever had friends, relatives or close acquaintances who are transgender? (N [%])*			
Yes			
No	9 (16.4%)	8 (14.3)	0.761^a^
	46 (83.6)	48 (85.7)	
*Have you ever treated transgender people? (N [%])*			
Yes	29 (52.7)	25 (47.2)	0.564^a^
No	26 (47.3)	28 (52.8)	
Missing = 3			
If yes, how many? (Mean ± SD)	3.45 ± 2.72	2.68 ± 2.5	0.287^b^
*Have you received training to treat the transgender population during medical studies or later after graduating from medical school? (N [%])*			
Yes			
No			
Missing = 1	11 (20.0)	51 (92.7)	0.644^c^
	44 (80.0)	4 (7.3)	
*Are you ready to treat a transgender patient? (N [%])*			
Yes	53 (96.4)	51 (92.7)	0.679^c^
No	2 (3.6)	4 (7.3)	
Missing = 1			
*When treating a transgender patient, will you treat him with the same attention and desire to help as any other patient? (N [%])*			
Yes			
No			
Missing = 1	53 (96.4)	51 (92.7)	0.679^c^
	2 (3.6)	4 (7.3)	
*Would you be interested in receiving training in the treatment of transgender patients? (N [%])*			
Yes			
No	38 (69.1)	41 (74.5)	0.525^a^
Missing = 1	17 (30.9)	14 (25.5)	

^a^Chi-square test, ^b^Mann–Whitney rank-sum test, ^c^Fisher exact test.

### Transgender Attitudes and Beliefs Scale Among Physicians

The reliability estimate for each of the subscales of the Hebrew version of the TABS questionnaire was high: *ω* = 0.95 for “Interpersonal comfort,” *ω* = 0.87 for “Sex/gender beliefs” and *ω* = 0.93 for “Human value,” showing high internal consistency for each subscale. The values of subscale correlations among the three subscales showed higher correlation between the “Interpersonal comfort” and “Sex/gender beliefs” subscales (*r* = 0.92), than between “Interpersonal comfort” and “Human value” (*r* = 0.31), and “Sex/gender beliefs” and “Human value” (*r* = 0.31) subscales.

There were differences in the “Interpersonal comfort” and “Sex/gender beliefs” subscales of the TABS questionnaire between senior and resident physicians, with more favorable attitudes among senior physicians (Table 3). The mean (SD) total scores were 81.8 (18.8) and 69.7 (24.1) (*p* = 0.005) for senior physicians and residents, respectively, for the “Interpersonal comfort,” and 54.3 (12.6) and 45.5 (14.4) (*p* < 0.001) for the “Sex/gender beliefs” subscale. The score was also higher among senior physicians compared to residents for the “Human value” subscale at 32.0 (6.8) vs. 29.4 (8.5), but this difference did not reach statistical significance (*p* = 0.08).

**Table 3:** Transgender Attitudes and Beliefs Scale

Statement	Specialists M ± SD	Residents M ± SD	*p*-value^a^
Factor 1. Interpersonal Comfort			
1. I would feel comfortable having a transgender person into my home for a meal	5.56 ± 1.82	4.80 ± 2.18	**0.052**
2. I would be comfortable being in a group of transgender individuals	5.37 ± 1.81	4.44 ± 2.33	**0.023**
R. 3. I would be uncomfortable if my boss was transgender	6.00 ± 1.77	4.96 ± 2.22	**0.008**
R. 4. I would feel uncomfortable working closely with a transgender person in my workplace	6.15 ± 1.85	5.50 ± 2.01	0.084
5. If I knew someone was transgender, I would still be open to forming a friendship with that person	5.57 ± 2.17	4.98 ± 2.23	0.164
6. I would feel comfortable if my next-door neighbor was transgender	6.19 ± 1.42	5.19 ± 2.06	**0.004**
7. If my child brought home a transgender friend, I would be comfortable having that person in my home	5.67 ± 1.81	4.32 ± 2.30	**0.001**
R. 8. I would be upset if someone I'd known for a long time revealed that they used to be another gender	5.76 ± 1.59	4.85 ± 2.33	**0.02**
R. 9. If I knew someone was transgender, I would tend to avoid that person	6.13 ± 1.85	5.20 ± 2.24	**0.021**
R. 10. If a transgender person asked to be my housemate, I would want to decline	5.11 ± 2.07	4.60 ± 2.26	0.228
R. 11. I would feel uncomfortable finding out that I was alone with a transgender person	5.93 ± 1.79	5.53 ± 2.02	0.283
12. I would be comfortable working for a company that welcomes transgender individuals	6.06 ± 1.65	5.19 ± 1.98	**0.015**
R. 13. If someone I knew revealed to me that they were transgender, I would probably no longer be as close to that person	5.94 ± 1.69	4.79 ± 2.13	**0.003**
R. 14. If I found out my doctor was transgender, I would want to seek another doctor	6.33 ± 1.71	5.37 ± 2.15	**0.011**
Overall score of the Factor 1—Interpersonal Comfort	81.76 ± 18.75	69.71 ± 24.05	**0.005**
Average score of the Factor 1—Interpersonal Comfort	5.84 ± 1.34	4.98 ± 1.72	**0.005**
Factor 2. Sex/Gender Beliefs			
R. 1. A person who is not sure about being male or female is mentally ill	6.31 ± 1.33	5.55 ± 1.91	**0.018**
2. Whether a person is male or female depends upon whether they feel male or female	5.15 ± 2.12	4.19 ± 2.03	**0.018**
R. 3. If you are born male, nothing you do will change that	5.30 ± 2.10	4.43 ± 2.22	**0.041**
R. 4. Whether a person is male or female depends strictly on their external sex-parts	6.13 ± 1.44	5.04 ± 2.03	**0.002**
R 5. Humanity is only male or female; there is nothing in between	5.33 ± 2.23	4.17 ± 2.22	**0.008**
6. If a transgender person identifies as female, she should have the right to marry a man	5.64 ± 1.73	4.77 ± 2.17	**0.025**
7. Although most of humanity is male or female, there are also identities in between	5.33 ± 1.99	4.22 ± 2.11	**0.006**
8. R. All adults should identify as either male or female	5.06 ± 2.14	4.04 ± 2.25	**0.018**
9. R. A child born with ambiguous sex-parts should be assigned to be either male or female	5.0 ± 2.10	4.10 ± 2.16	**0.031**
10. A person does not have to be clearly male or female to be normal and healthy	5.20 ± 2.27	4.94 ± 2.06	0.537
Overall score of Factor 2—Sex/Gender Beliefs	*54.33* ± *12.62*	*45.53* ± *14.42*	** < *0.001***
Average score of Factor 2—Sex/Gender Beliefs	*5.43* ± *1.26*	*4.55* ± *1.44*	** < *0.001***
Factor 3. Human value			
1. Transgender individuals are valuable human beings regardless of how I feel about transgenderism	6.44 ± 1.41	5.85 ± 1.88	0.067
2. Transgender individuals should be treated with the same respect and dignity as any other person	6.49 ± 1.41	6.08 ± 1.69	0.169
3. I would find it highly objectionable to see a transgender person being teased or mistreated	6.46 ± 1.31	5.75 ± 2.05	**0.036**
4. Transgender individuals are human beings with their own struggles, just like the rest of us	6.15 ± 1.76	5.73 ± 2.13	0.275
5. Transgender individuals should have the same access to housing as any other person	6.47 ± 1.46	6.0 ± 1.93	0.156
Overall score of Factor 3—Human Value	*32.00* ± *6.75*	*29.39* ± *8.49*	*0.08*
Average score of Factor 3—Human Value	*6.40* ± *1.35*	*5.88* ± *1.70*	*0.08*

Significant differences are emphasized with bold

R-reversed scale. The scores on the reversed scale questions are shown after transformation. ^a^Mann–Whitney rank-sum test

In the dichotomous division of the subscales, 60 (54.1%), 38 (34.2%), and 82 (73.9%) of all physicians scored favorably on “Interpersonal comfort,” “Sex/gender beliefs,” and “Human value” subscales, respectively. Senior physicians scored more favorably than residents on all the subscales (38 (69.1%) vs. 22 (39.3%), *p* = 0.004 for “Interpersonal comfort,” 24 (43.6%) vs. 14 (25.0%), *p* = 0.025 for “Sex/gender beliefs,” and 48 (87.3%) vs. 34 (60.7%), *p* = 0.006 for “Human value”).

### Factors Associated With Favorable Attitudes and Beliefs Toward Transgender People

The multivariable logistic regression models on the association of various factors with a favorable attitude toward transgender people in the subscales of the TABS questionnaire are presented in Table 4.

**Table 4:** Factors associated with favorable attitude on TABS questionnaire

Variable	OR	95% CI	*P*	OR	95% CI	*p*	OR	95% CI	*p*
	Interpersonal comfort			Sex/gender beliefs			Human value		
Age	0.99	0.94–1.04	0.681	0.97	0.91–1.03	0.322	0.95	0.89–1.01	0.087
Sex (female)	1.95	0.81–4.82	0.139	1.52	0.60–3.94	0.385	**4.03**	**1.36–13.69**	**0.016**
Ethnicity (non-Jewish)	**0.26**	**0.09–0.78**	**0.017**	0.79	0.23–2.72	0.703	0.315	0.08–1.12	0.077
Seniority (resident)	0.62	0.19–1.98	0.420	0.38	0.09–1.38	0.151	**0.192**	**0.04–0.85**	**0.038**
Level of religiosity (religious to a large or significant extent)	**0.34**	**0.13–0.85**	**0.022**	**0.11**	**0.03–0.33**	**0.000**	0.628	0.19–2.10	0.438

Significant variables are highlighted in bold

TABS: Transgender Attitudes and Beliefs Scale, OR: odds ratio, 95% CI: 95% confidence interval

Physicians of non-Jewish ethnicity were less likely to have a favorable score on the “Interpersonal comfort” domain at aOR = 0.26 (0.09–0.78), as were those with a high level of religiosity at aOR = 0.34 (0.13–0.85). Physicians with a high level of religiosity were also less likely to have favorable “Sex/gender beliefs” at OR = 0.11 (0.03–0.33). Female sex was positively associated with favorable “Human value” regarding transgender people at OR = 4.03 (1.36–13.69) and being a resident was negatively associated at aOR = 0.19 (0.04–0.85).

## Discussion

One of the central findings of our study is that family physicians in southern Israel acknowledged the human value of transgender people, with 74% of the physicians having a favorable score on the “Human value” of transgender people. At the same time, the percent of those who scored favorably on the “Interpersonal comfort,” and the “Sex/gender beliefs” scale was low at 54% and 34%, respectively. These discrepancies among responses to the different subscales could signify the commitment of physicians to basic human and medical professional values on the one hand, and rooted prevalent stereotypes on sex and gender on the other. These stereotypes are prevalent among physicians from other countries (Madera et al., 2019). The mean scores in the subscales of TABS were comparable to those reported in the general population of the USA (Kanamori et al., 2017), but lower than those reported among physicians in central Israel (Landau et al., 2020; Tepp et al., 2018). Compared to the southern region, the central region, where those studies were carried out, is characterized by a higher socioeconomic level and a lower level of religiosity (OECD, 2020).

The main aim of the present study was to compare attitudes toward transgender people between senior physicians and residents in family medicine. More senior physicians than residents exhibited favorable attitudes toward transgender people in all the subscales of the TABS questionnaire when divided dichotomously, and in two subscales (“Interpersonal comfort” and “Sex/gender beliefs”) when the mean values of these subscales were compared. To interpret the differences in the attitudes toward transgender people between senior physicians and residents, we began with differences in the baseline characteristics of these groups. The differences in age and physician seniority are easily explained. Other important background differences were the higher percent of physicians who were born in Israel and the lower percent of physicians of Jewish ethnicity among residents. This demographic shift among the physicians is explained by the fact that the older generation of physicians in Israel was largely comprised of immigrants from the former USSR, whereas many residents are native-born, including a high percent of Arab residents, who completed their medical studies abroad (Treister-Goltzman & Peleg, 2023). It is likely that this demographic diversity led to significant differences in the religiosity level between the study groups, with more than twice as many residents as senior physicians reporting that religion guided them in everyday life. Indeed, when adjusted for other background variables in the multivariable model, being a resident, as opposed to a senior physician, was found to have a significant association only with the “Human value” domain, decreasing the odds of a favorable attitude. On the contrary, in studies from central Israel (Landau et al., 2020; Tepp et al., 2018), being a senior physician (pediatrics and internal medicine) significantly increased the adjusted odds for unfavorable attitudes in the “Interpersonal comfort” domain, and very slightly increased the odds for an unfavorable attitude in the “Sex/gender beliefs” domain. Studies from other countries also showed that more experienced senior physicians had less favorable attitudes toward transgender people (Feroe et al., 2022), although among psychiatrists in the USA, senior physicians showed more positive attitudes (Ali et al., 2016). Another factor positively associated with favorable attitudes in the “Human value” subscale was female gender. Previous studies have also shown more favorable attitudes toward transgender individuals among female physicians than males (Landau et al., 2020; Rowan et al., 2019). In the “Interpersonal comfort” domain, non-Jewish ethnicity was associated with a less favorable attitude. The Muslim population of Israel is characterized by more conservative religious beliefs and cultural norms (Pew Research Center, 2016). While the study among internal medicine and family physicians from central Israel did not assess the adjusted association of ethnicity with attitudes toward transgender people, it did find significantly lower scores for all three TABS subscales for non-Jewish physicians (Tepp et al., 2018). The inverse relationship between the level of religiosity and favorable “Interpersonal comfort” and “Sex/gender beliefs” domains was like the results of studies from central Israel (Landau et al., 2020; Tepp et al., 2018). Although studies conducted around the world did not use the same measurement tool to assess attitudes toward transgender people, they also found a negative association between religiosity and attitude toward transgender people (Feroe et al., 2022; Sharma et al., 2019).

There were no differences in personal and professional experience with transgender people and more than half of the physicians from both groups reported having treated transgender patients. Studies conducted around the world showed that more than two-thirds of physicians had prior professional experience treating transgender people (Bashir et al., 2023; Campbell et al., 2019). In contrast, only about 20% of physicians in both groups received professional training on transgender health care. This number is very low compared to the developed world, with more than half of the physicians in the USA (Jewell & Petty, 2024; Shires et al., 2018) and Britain (Dubin et al., 2018), reporting that they had undergone training on transgender health, although they rated this training as insufficient (Dubin et al., 2018; Jewell & Petty, 2024; Shires et al., 2018). More than 90% of the physicians in our study were interested in receiving such training and expressed a professional commitment to treat transgender patients as any other patient. This finding is consistent with other studies around the world, where physicians were willing to treat transgender people and to improve their knowledge on transgender health (Barber et al., 2023; Do & Nguyen, 2020; Lu et al., 2022; Shires et al., 2018).

The findings of the present study, as well as of other studies carried out in Israel and abroad, could facilitate the development of curricula on transgender care in medical education. An excellent recent systematic review showed that the main barriers for implementing such a curriculum are lack of educational materials, lack of faculty expertise, time/costs constraints, and challenges in recruiting and compensating transgender guest speakers (van Heesewijk et al., 2022). Facilitators included scaffolding learning throughout the curriculum, drawing on the expertise of transgender people and engaging learners in skills-based training (van Heesewijk et al., 2022). In the Israeli context, with the described demographic diversity of physicians, the first steps in developing such a curriculum might be adopted from the Queen’s University Internal Medicine residency program (Sandre et al., 2025). In their case, the faculty followed Kern’s approach to curriculum design, which starts with recognizing a gap in knowledge, attitudes, and skills, and a focused needs assessment based on interviews focused on the specific needs of learners (Sandre et al., 2025).

### Strengths and Limitations

The first limitation of our study is its questionnaire-based design with possible recall/social desirability/habituation biases. Second, it was difficult to make comparisons with other reports in the international literature because different instruments were used in the studies, although a few of the other studies (Kanamori et al., 2017; Landau et al., 2020; Tepp et al., 2018) did use the same TABS tool. The cross-sectional design assessed associations at a single point in time only, rather than causal or temporal relationships between variables. However, questionnaires are still the most acceptable and feasible way to assess the attitudes of physicians. Finally, the regional nature of the study, with a specific demographic profile of physicians in the peripheral southern region of Israel, limits the generalizability of the findings.

The strengths of this study include the adequate representation of family medicine senior physicians and residents, and the use of a comprehensive questionnaire that assessed multiple personal and professional characteristics, as well as multiple domains of attitudes toward transgender people, enabling us to highlight problematic issues.

### Conclusion

Family physicians in southern Israel showed that they appreciate “Human value” in transgender people, but scored low on the “Interpersonal comfort” and “Sex/gender beliefs” domains. They expressed a desire to improve their level of knowledge on transgender care, which should be addressed by policy makers in undergraduate and continuous medical education. Our study identified physician characteristics associated with a less favorable attitude, such as male sex and a high level of religiosity, suggesting a need to focus on these groups of physicians for further training.

## Data Availability

The data that support the findings of this study are available from the corresponding author, YTG, upon reasonable request.

## Code Availability

The code that supports the findings of this study is available from the corresponding author, YTG, upon reasonable request.
